# Dexamethasone Suppression Test May Predict More Severe/Violent Suicidal Behavior

**DOI:** 10.3389/fpsyt.2020.00097

**Published:** 2020-03-04

**Authors:** Adrián Alacreu-Crespo, Emilie Olié, Sebastien Guillaume, Chloé Girod, Aurélie Cazals, Isabelle Chaudieu, Philippe Courtet

**Affiliations:** ^1^ PSNREC, Univ. Montpellier, INSERM, CHU de Montpellier, Montpellier, France; ^2^ Department of Emergency Psychiatry and Acute Care, Lapeyronie Hospital, CHU Montpellier, Montpellier, France; ^3^ FondaMental Foundation, Créteil, France; ^4^ Univ. Montpellier, Inserm, Neuropsychiatry: Epidemiological and Clinical Research, Montpellier, France

**Keywords:** dexamethasone suppression test, salivary cortisol, hypothalamic-pituitary-adrenal axis, suicide attempt, severity, intent

## Abstract

**Introduction:**

Several studies demonstrated that the hypothalamic-pituitary-adrenal (HPA) axis is dysregulated in suicide attempters. Prospective studies found that people with an abnormal response at the dexamethasone suppression test (DST) are more likely to commit suicide. However, whether DST may predict suicide attempts remains less clear. A possible strategy to address this question is to consider the suicide attempt lethality.

**Objectives:**

(1) To compare the pre- and post-DST cortisol levels in serious/violent suicide attempters and in non-serious/non-violent suicide attempters, and (2) to investigate whether cortisol level can predict new suicide attempts or their lethality.

**Methods:**

The study included 70 recent suicide attempters (25 with a serious or violent attempt) who were followed for two years. Three saliva samples for cortisol measurement were obtained at 8a.m., 3p.m., and 9p.m. before the DST (pre-DST). Then, at 11 p.m., 1 mg of dexamethasone was given orally. The following day (post-DST), three saliva samples were collected at the same hours as before. The post-DST–pre-DST salivary cortisol Δ index was calculated for each collection time. The Risk-Rescue Ratio Scale (RRRS) and the Suicidal Intent Scale (SIS) were used to characterize the suicide attempt at inclusion and those occurring during the follow-up.

**Results:**

Post-DST cortisol level at 9 p.m. was higher in patients with an initial violent or serious suicide attempt than in non-violent/non-serious attempters (p < .010). Higher post-DST cortisol at 9p.m. was associated with lower RRRS rescue score and higher clinical impression of suicide severity at inclusion. Among the 66 patients who completed the follow-up, 26 attempted suicide again at least once. Higher pre-DST cortisol at 8a.m. predicted new suicide attempts during the follow-up (OR = 2.15 [1.11, 4.15]), and higher cortisol Δ index at 9p.m. was associated with higher suicide intent during the follow-up.

**Conclusions:**

Our results suggest that HPA axis hyper-reactivity monitored with the DST is a marker of violent/serious suicide attempt with lower rescue possibility. Furthermore, higher changes between pre-DST and post-DST cortisol levels may predict higher suicide intent. These findings might help to characterize the biological features of nearest suicide phenotypes.

## Introduction

Suicide is one of the leading causes of death worldwide (1). Therefore, the causes and risk factors that lead people to commit suicide must be identified for improving suicide prevention. However, it is difficult to predict suicide because many factors involved in suicidal behavior interact with each other. According to the stress-diathesis model, suicidal behavior is the result of the interaction between acute or chronic stress factors and traits of susceptibility to commit suicide (diathesis) (2). It has been suggested that some diathesis biomarkers combined with severe stressful events could be a predictor of suicide risk, independently of psychiatric comorbidity (3). The glucocorticoid cortisol, the “stress” hormone implicated in metabolic, cognitive and inflammatory processes, is among these potential diathesis biomarkers (4). Cortisol is secreted from the adrenal glands upon activation of the hypothalamic-pituitary-adrenal (HPA) axis to increase access to energy to face a stressor (5). Elevated cortisol levels have been associated with impairment of some cognitive functions involved in suicide risk, such as cognitive control, emotional/social processing, and decision-making (6, 7). Moreover, stress-induced continuous or repetitive liberation of cortisol will damage the body, including the HPA axis (8).

The Dexamethasone Suppression Test [DST: (9)] is classically used to test HPA axis function. In basal conditions, cortisol level shows a circadian rhythm with a peak of release in the morning at wake-up time. Then, it decreases progressively during the day until early night (between 6 p.m. and 10 p.m.). In the DST, cortisol level is quantified the day following the oral administration of 1 mg of the synthetic corticoid dexamethasone. Dexamethasone is supposed to inhibit cortisol release, inducing a more pronounced decrease during the day^1^. Cortisol inhibition failure (i.e., non-suppression) is considered a marker of HPA axis hyperactivity. A meta-analysis of DST responses in patients with depression found that non-suppression was associated with subsequent completed suicides (11). This finding was replicated in some follow-up studies (12–14), whereas other works found that non-suppression was related to suicide completion only in elderly patients (15) and in men (16–18). Despite these discrepancies, the most recent literature review on this topic clearly shows that DST is a robust marker of suicide completion in inpatients with depression and history of suicidal behavior (19). On the other hand, the relationship between DST and suicide attempts is less consistent (20). Previous studies showed that DST non-suppression was related to suicide attempt in young individuals (21) and to high scores in scales that quantify the risk of future attempts (22). Other prospective reports showed non-significant relationships between non-suppression and suicide attempts (23, 24).

These discrepancies could be explained by differences among suicide attempters, particularly in the potential lethality of the used method, the choice of a violent method, the medical consequences of the suicidal act, and the level of suicidal intent (25, 26). Serious and violent suicide attempters might show different psychological features compared with non-violent/non-serious suicide attempters (27). In a cluster analysis of 1,009 suicide attempters, Lopez-Castroman et al. (28) identified a cluster of individuals with more violent or severe attempts, higher number of attempts, and earlier age at first attempt. Moreover, Giner et al. (29) showed that violent/serious attempters (i.e., at higher risk for complete suicide) are more likely to be suicide repeaters, with higher suicide lethality than non-violent/severe attempters. Interestingly, Roy (30) found that post-DST cortisol level is higher in violent than in non-violent suicide attempters. Similarly, non-suppression has been significantly related to serious suicide attempts (23, 30, 31). Conversely, Lindqvist et al. (32) reported a negative correlation between post-DST cortisol level and current suicidal intent in patients with major depression.

Therefore, more research is needed to test whether DST is a marker of future suicide attempts in patients with depression and history of suicide. Based on previous findings (23, 30, 31), we hypothesized that post-DST cortisol level might help to identify violent and serious attempters, and predict the lethality of a new suicide attempt. To test this hypothesis, we performed DST in recent suicide attempters who were prospectively followed for 2 years. We wanted to (1) compare pre-DST and post-DST cortisol levels between violent/serious and non-violent/non-serious suicide attempters, and (2) investigate whether cortisol level may predict new suicide attempts and/or their lethality.

## Methods

### Subjects

**Figure 1:**
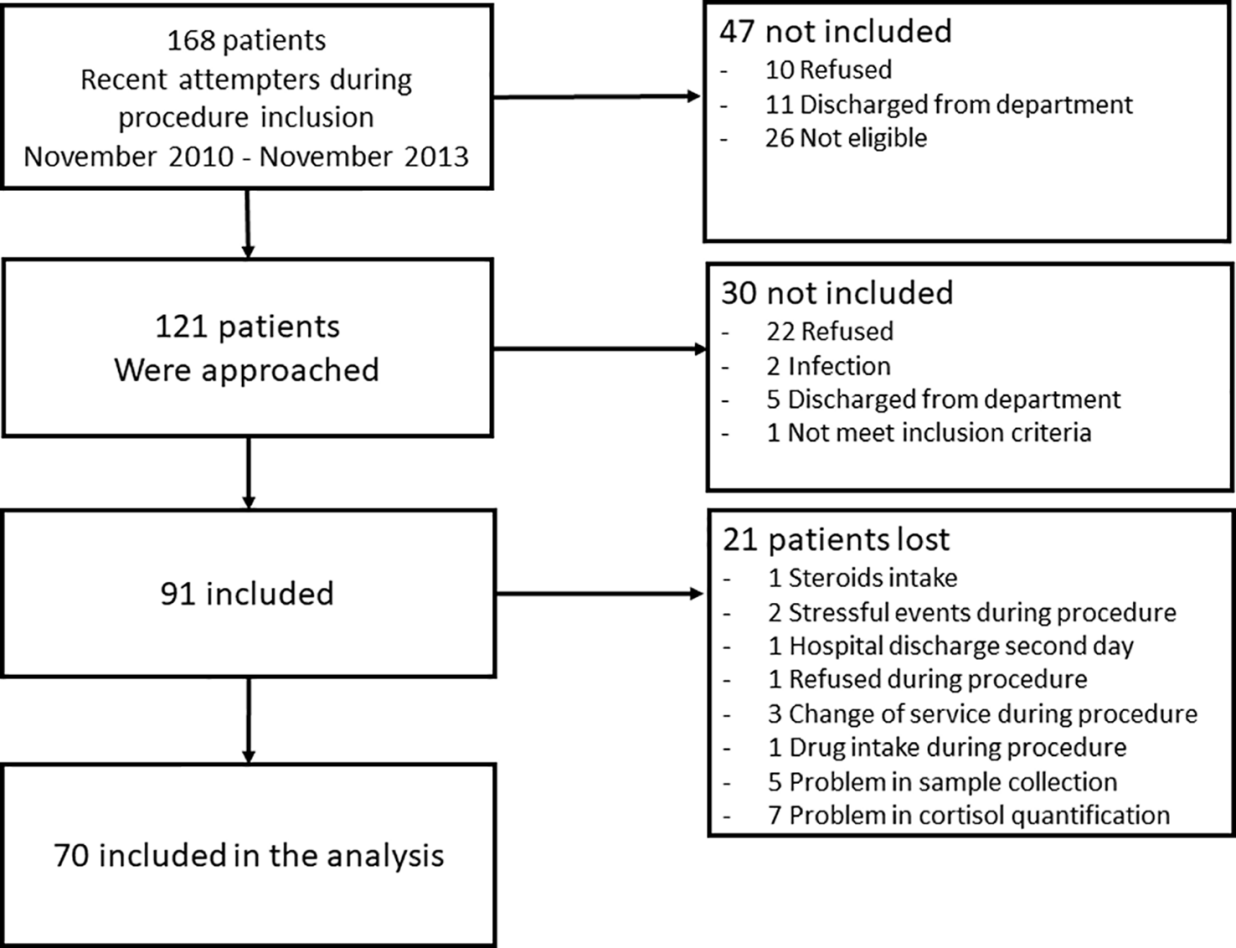
Flow chart of the study recruitment process.

This study included 70 adult inpatients (22 men and 48 women, mean age ± SD = 41.59 ± 11.86 years) admitted to a specialized unit at Montpellier University Hospital, France. Patients were referred to this unit from the emergency room or another clinical department after a suicide attempt (i.e., a self-damaging act carried out with at least some intention to die). They were all recent suicide attempters (no more than 1 week between the attempt and DST administration). Twenty-five patients were serious or violent attempters. Violent suicide attempts were classified using the criteria of Asberg and colleagues (25): hanging, drowning, jumping from heights, and use of firearms or knives. A serious suicide attempt was defined as a self-damaging act committed using a non-violent method that required hospitalization in intensive care (33). A list of the suicide attempt methods used by the included patients can be found in **Table 1**. All patients had a current major depressive episode. Inclusion criteria were: hospitalization for suicide attempt, current major depressive episode as main diagnosis, and being older than 18 years of age. Exclusion criteria were: pregnant or breastfeeding woman, current treatment or medical condition known to interfere with the DST results (e.g., Cushing’s syndrome or corticosteroid intake), and lifetime diagnosis of schizoaffective disorder or schizophrenia. **Figure 1** show a flow chart of recruitment process.

**Table 1:** List of suicide attempt methods at baseline.

Method of suicide	Number of patients
Medical intoxication	N = 45
Medical intoxication (serious)	N = 12
Drowning	N = 5
Hanging	N = 3
Cutting	N = 3
Gun shooting	N = 1
Jumping	N = 1

The *a priori* power and sample size estimation based on Roy et al. (30) indicated that 10 participants were needed to obtain a significant between-group difference for cortisol level, with alpha significance level fixed at 0.05 and statistical power of 0.80.

### Procedure

The study protocol was approved by the local research ethics committees (CPP Montpellier Sud-Méditerranée IV, CHU Montpellier) and performed according to the tenets of the Declaration of Helsinki. All participants signed a written informed consent.

At inclusion, patients who met the inclusion criteria and accepted to participate in the study were hospitalized for two days during which they had a clinical examination before the DST. Follow-up visits were scheduled at 6, 12, 18, and 24 months after the DST. The post-DST suicide history (i.e., new suicide attempts or suicide) was obtained during these visits or by telephone calls. The death certificate was consulted for patients who died during the follow-up. For patients lost to follow-up, information on new suicide attempt(s) was collected from the medical records of the Montpellier University Hospital emergency department (the only emergency unit accredited to manage suicide attempts in the area) for the 2 years after the inclusion date in order to have the same follow-up period for all patients (data were missing for four patients).

### Clinical Assessment

Socio-demographic variables: At inclusion, socio-demographic variables (demographic characteristics, education level, employment status, marital status, gynecological history, medication and smoking history) were collected during an interview. Height and weight were also measured.

Psychiatric comorbidity: Psychiatric disorders and suicidal behavior were assessed by senior psychiatrists using the French version of the Mini International Neuropsychiatric Interview [MINI 5.0: (34)].

Depression: Depressive symptomatology was evaluated using the 17-item Hamilton scale (35).

Suicide intent: The suicide attempt intent was evaluated with the Suicidal Intent Scale [SIS: (36)]. This scale includes 15 items: the first eight items evaluate the circumstances of the act, and the last seven items assess the feelings and thoughts at the time of the act.

Suicide lethality: The suicide attempt lethality was evaluated using the Risk-Rescue Rating Scale [RRRS: (37)]. This scale includes 10 items to evaluate the suicide lethality, defined as the probability of inflicting irreversible damage, in function of five risk factors (method, impaired consciousness, toxicity, reversibility, and treatment required), and five rescue factors (location, person initiating rescue, probability of discovery, accessibility to rescue, and time until discovery). A senior psychiatrist evaluated the clinical general impression (CGI) of the suicide attempt severity using a scale from 1 (Minimal) to 4 (Extreme).

At inclusion, the SIS, RRRS and CGI evaluations concerned the most recent suicide attempt. During the follow-up interviews, the SIS and RRRS were evaluated retrospectively if a participant attempted suicide between follow-up visits. This could be done for 12 patients. Information on the CGI score was obtained for 17 re-attempters from the emergency department files. If a patient attempted suicide two or more times during the follow-up, only the data concerning the most severe attempt were used for the analysis.

### Salivary Cortisol Quantification

Detailed written and verbal instructions for saliva sample collection were given to all patients. They were instructed to avoid eating or drinking anything (only water), smoking or brushing their teeth at least 1 h prior to sample collection. Nurses made sure that patients did not smoke or take alcohol, and did not eat or drink 2 h before each salivary sample collection. They also monitored the adherence to the saliva collection times and dexamethasone intake. Saliva was collected using Salivette^®^ tubes (Stardex) at 8 a.m., 3 p.m., and 9 p.m. before (pre-DST) and after (post-DST) the oral intake of 1 mg of dexamethasone at 11 p.m. Samples were centrifuged (5,000 rpm, 15 + 2°C) and stored at −20°C until analysis.

Salivary cortisol levels were determined with the Spectria Cortisol radioimmunoassay (RIA) Kit (Orion Diagnostica, Espoo, Finland) or by electrochemiluminescence (ECLIA) with the Elecsys Cortisol II Kit from Cobas^®^. All cortisol samples (pre-DST and post-DST) from the same patient were analyzed using the same method. The intra- and inter-assay variation coefficients were all below 9.3%. Cortisol scores were standardized separately for the two analysis methods (RIA and ECLIA) by converting each participant’s raw scores to Z-scores. This statistical procedure was previously used to combine data for populations with great differences in basal endocrine measures, for example sex differences in testosterone level (38).

As the pre-DST cortisol levels might affect the post-DST cortisol response, a delta index (Δ) was calculated for the post-DST and pre-DST values at the same hour (e.g., post-DST 8a.m. – pre-DST 8a.m.). Lower δ values reflect a decrease of post-DST cortisol levels relative to the pre-DST levels.

### Data Analysis

The presence of outliers was checked using the ± 3 standard deviation criterion. The Kolmogorov-Smirnov test was used to check the normal distribution of variables. Variables not normally distributed were normalized with the log10 method. Then, preliminary analyses were performed using the Student’s t and chi-square tests to identify differences in sociodemographic and clinical variables between serious/violent and non-serious/non-violent suicide attempters at inclusion, and between individuals that attempted or not suicide during the follow-up. Each pre- and post-DST cortisol value and Δ index were correlated with the sociodemographic and clinical variables, using Pearson correlations for continuous variables and Kendall τ for dichotomic variables.

Next, to assess the DST response in function of the violence or severity of the initial suicide attempt, two repeated-measures ANOVA analyses were performed using the pre-DST and post-DST cortisol values, respectively, with “violent/serious suicide attempt” (Yes/No) as between-subject factor, and “hour” (8 a.m., 3 p.m., and 9 p.m.) as within-subject factor. To reduce the likelihood of type I error, the degree of freedom was adjusted with the Greenhouse-Geisser correction (39), when required. Then, ANOVA analysis was performed using the `violent/serious suicide attempt´ (Yes/No) factor and the cortisol Δ indexes. All analyses were adjusted for sociodemographic or clinical variables when preliminary analyses showed between-group differences. Post-hoc tests were performed with simple contrasts using the Bonferroni correction.

Multivariate logistic regressions were used to predict new suicide attempt(s) after adjusting for sociodemographic or clinical variables that were significantly different between the with and without new suicide attempt(s) groups. Each pre-DST, post-DST cortisol, and Δ index value was used as predictor.

Finally, the relationships between pre-DST, post-DST cortisol, and Δ index values and CGI, RRRS and SIS scores at inclusion and during the follow-up were investigated using the Pearson correlation method. In the case of significant relationships, regression models were used after adjusting for sociodemographic and clinical variables that were significantly correlated with cortisol measures. Moreover, analyses were always adjusted for mood stabilizer intake, because previous studies showed that mood stabilizers are a confounder for cortisol (40, 41).

The alpha significance level was fixed at 0.05. Partial eta squared was reported for ANOVA and ANCOVA as a measure of the effect size. β − 1 was reported as a measure of the a posteriori statistical power. Data is available in **Supplementary Material**. All statistical analyses were performed with SPSS 20.0.

## Results

### Correlations Between Sociodemographic/Clinical Variables and Cortisol Values

Clinical and sociodemographic variables at inclusion were not significantly different between violent/serious (n=25) and non-violent/non-serious suicide attempters (n=45) (all p > .05; **Table 2**). The correlations between pre- and post-DST cortisol values and sociodemographic and clinical variables are presented in **Table 3**.

**Table 2:** Sociodemographic and clinical variables at inclusion.

	Violent/Serious SA		Reattempt during follow-up		p
	Yes	No	Yes	No
N =	25	45	26	40	
Age	42.27 ± 2.09	40.86 ± 1.89	36.28 ± 2.29	43.61 ± 1.79	*Reattempt = p* < .014
Education (years)	14.00 ± .58	12.70 ± .34	13.16 ± .52	13.30 ± .39	NS
BMI	22.45 ± .73	22.59 ± .66	22.82 ± .97	22.49 ± .56	NS
HAMD score	13.52 ± 1.17	13.96 ± .81	14.12 ± 1.35	13.08 ± .75	NS
Women, n (%)	15 (60.0%)	32 (72.7%)	17 (68.0%)	27 (67.5%)	NS
Current smoker, n (%)	12 (48.0%)	21 (47.7%)	13 (52.0%)	18 (45.0%)	NS
Separate/Divorced, n (%)^1^	9 (36.0%)	14 (31.8%)	8 (32.0%)	13 (32.5%)	NS
Substance abuse, n (%)	7 (28.0%)	11 (25.0%)	7 (28.0%)	11 (27.5%)	NS
Alcohol abuse, n (%)	7 (28.0%)	16 (36.4%)	9 (36.0%)	12 (30.0%)	NS
Anxiety disorder, n (%)	21 (84.0%)	37 (84.1%)	22 (88.0%)	33 (82.5%)	NS
Bipolar disorder, n (%)	9 (40.9%)	12 (27.9%)	8 (32.0%)	12 (32.4%)	NS
Eating disorder, n (%)	5 (20.0%)	10 (22.7%)	6 (24.0%)	9 (22.5%)	NS
PTSD, n (%)	8 (32.0%)	12 (27.9%)	8 (33.3%)	11 (27.5%)	NS
Fam. history of SA, n (%)	14 (58.3%)	23 (53.5%)	14 (60.9%)	22 (55.0%)	NS
Antidepressants, n (%)	14 (58.3%)	27 (61.4%)	14 (58.3%)	25 (62.5%)	NS
Anxiolytics, n (%)	15 (62.5%)	29 (65.9%)	15 (62.5%)	27 (67.5%)	NS
Antipsychotics, n (%)	17 (70.8%)	28 (63.6%)	15 (62.5%)	28 (70.0%)	NS
Mood stabilizers, n (%)	13 (54.2%)	15 (34.1%)	14 (58.3%)	13 (32.5%)	*Reattempt p < .*043
Lithium, n (%)	1 (4.2%)	1 (2.3%)	1 (4.2%)	1 (2.5%)	NS
Reg. mens. cycle, n (%)	7 (53.8%)	15 (68.2%)	12 (80.0%)	10 (52.6%)	NS

SA, Suicidal attempt; BMI, Body mass index; HAMD, 17-item Hamilton scale; PTSD, Post-traumatic stress disorder; Fam. history of SA, Family history of suicidal attempt; Reg. mens. cycle, Regular menstrual cycle.

^1^0 (Single) 1 (Married) 2 (Separated/Divorced).

**Table 3:** Bivariate correlations between cortisol measures and sociodemographic and clinical variables.

	Pre-DST C 8a.m.	Pre-DST C 3p.m.	Pre-DST C 9p.m.	Post-DST C 8a.m.	Post-DST C 3p.m.	Post-DST C 9p.m.	ΔC 8a.m.	ΔC 3p.m.	ΔC 9p.m.
Age	−.10	−.05	−.07	−.05	−.19	.06	.06	−.12	.13
Sex	−.03	−.10	−.001	−.01	−.06	−.13	.01	.12	−.19
Education (years)	−.22	.16	−.04	−.10	−.01	.07	.14	−.17	.11
Divorced/Sep.	.16	.11	.02	.05	−.04	.11	−.14	−.23^*^	.11
BMI	−.08	−.11	−.17	.11	−.06	−.05	.13	.02	.18
No Cigarettes/day	.04	.06	.05	.01	−.12	.03	−.04	−.04	−.02
HAMD score	.10	−.05	−.08	.003	−.05	−.07	−.09	.12	−.001
Substance abuse	.15	−.04	.07	.02	.12	.07	−.17	.003	.01
Alcohol abuse	−.02	−.12	−.05	−.06	−.03	−.19	−.03	.14	−.18
Anxiety disorder	.04	−.01	.02	−.04	−.06	.14	−.03	−.01	.11
Bipolar disorder	−.04	−.05	.09	.12	.03	.05	.13	.07	−.10
Eating disorder	.01	−.15	.02	−.15	−.05	−.01	−.10	.12	−.06
PTSD	.13	.01	.08	.08	.05	.12	−.12	−.04	−.001
Fam.History of SA	.29^**^	−.04	.20	.12	.13	−.05	−.17	.09	−.16
Antidepressants	−.11	.01	−.19	−.02	−.07	−.07	.07	−.02	.15
Anxiolytics	−.11	−.17	−.26^*^	−.13	−.15	−.16	.01	.05	.12
Antipsychotics	.01	.05	.001	.17	−.002	.15	.09	−.05	.17
Mood stabilizers	.17	.07	.31^**^	.12	.21^*^	.11	−.04	.14	−.22^*^
Lithium	−.07	.07	.06	.06	.09	.05	.09	−.13	−.05
Reg. mens. cycle	.17	.03	.08	−.05	−.04	.15	−.12	.11	.02

Pre-DST, Pre-dexamethasone; C, Cortisol; Post-DST, Post-dexamethasone; Sep, Separated; BMI, Body mass index; HAMD, 17-item Hamilton scale; PTSD, Post-traumatic stress disorder; Fam. History of SA, familial history of suicide attempt; Reg. mens. cycle, Regular menstrual cycle.

*p < .05, **p < .01.

### Association Between DST Response and Violent/Serious Suicide Attempt

**Figure 2:**
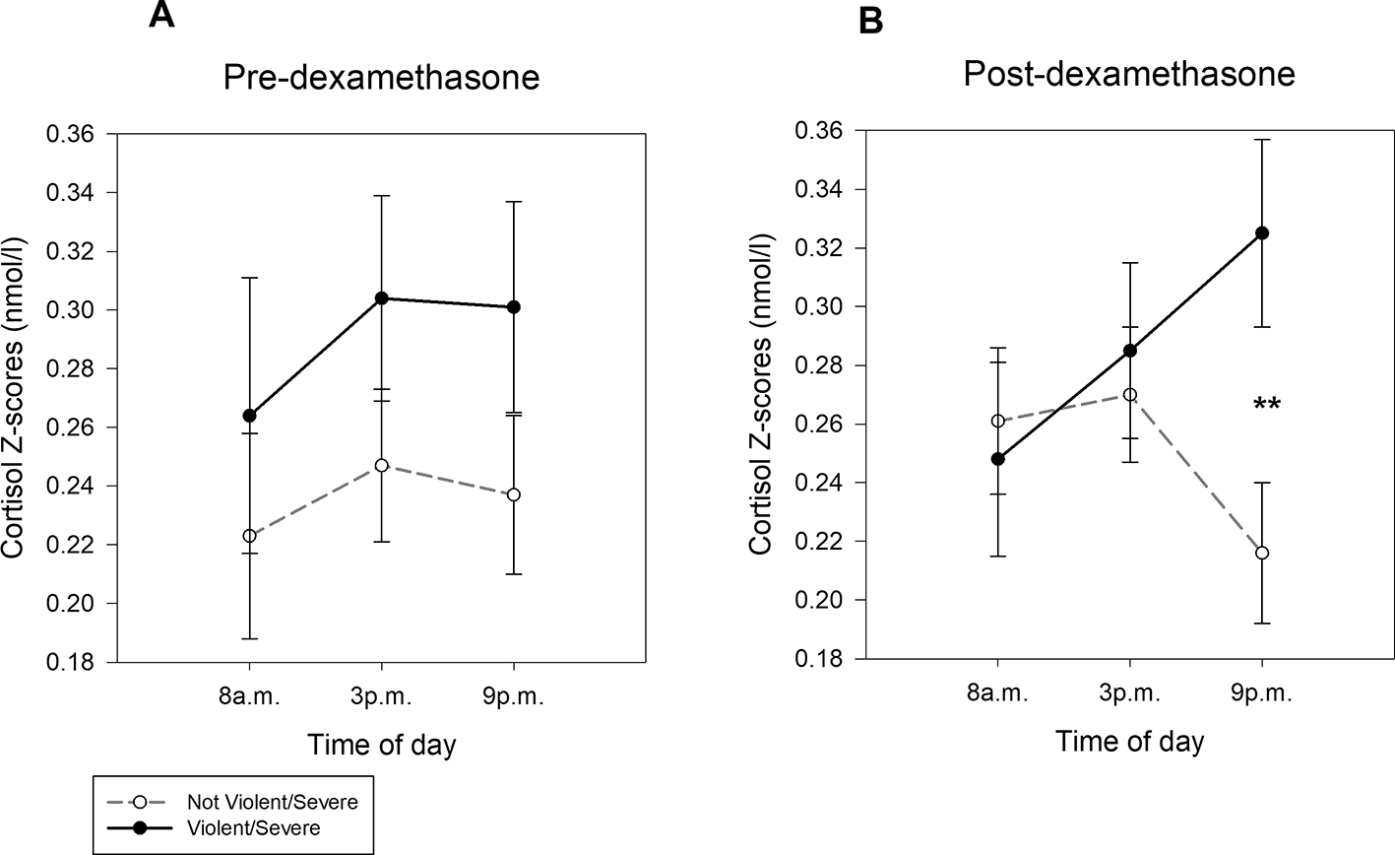
Cortisol levels (mean ± SEM of Z-scores) at 8a.m., 3p.m. and 9p.m. A) before and B) after oral administration of dexamethasone in saliva samples from violent/serious suicide attempters and non-violent/serious suicide attempters. **p < .010.

Analysis of the DST response in function of the violence or severity of the initial suicide attempt (‘violent/serious suicide attempt´ and “violent/serious suicide attempt” × “hour” interaction) found significant differences only for the post-DST cortisol values (“violent/serious suicide attempt” × “hour” interaction: F_1.6, 91.5_ = 8.29, p < .001, η^2^_p_ =.12, power =.92), but not for the pre-DST values (p > .05) (**Figure 2**). Post-hoc comparisons highlighted significant differences in the slopes of the violent/serious (F_2, 58_ = 3.67, p < .032, η^2^_p_ =.12, power =.68) and non-violent/non-serious suicide attempters (F_2, 58_ = 3.87, p < .027, η^2^_p_ =.11, power =.65). Moreover, post-hoc comparisons showed significantly higher post-DST cortisol levels at 9p.m. in the violent/serious suicide attempter group than in the non-violent/non-serious suicide attempter group (F_1, 59_ = 7.19, p < .010, η^2^_p_ =.11, power =.75; **Figure 2B**).

Finally, ANOVA did not show any significant difference in the cortisol Δ indexes between groups (all hours; p > .05).

### Associations Between DST Response and Characteristic of the Initial Suicide Attempt

The rescue sub-score of the RRRS was negatively associated with the pre-DST cortisol values at 3 p.m. (r = −.26, p < .046) and 9 p.m. (r = −.33, p < .011), and with the post-DST cortisol values at 8 a.m. (r = −.28, p < .031) and 9 p.m. (r = −.36, p < .005). Conversely, the CGI score (severity of suicide attempt) was positively associated with the pre-DST (r =.24, p < .049) and the post-DST cortisol values at 9 p.m. (r =.34, p < .005) (**Table 4**).

**Table 4:** Bivariate correlations between cortisol measures and suicidal risk and lethality at inclusion and during the follow-up.

	Pre-DST C 8a.m.	Pre-DST C 3p.m.	Pre-DST C 9p.m.	Post-DST C 8a.m.	Post-DST C 3p.m.	Post-DST C 9p.m.	ΔC 8a.m.	ΔC 3p.m.	ΔC 9p.m.
*Inclusion*									
RRRS Ratio	−.07	.16	.07	.07	.03	.19	.11	−.13	.13
Risk	−.06	−.01	.01	−.17	−.16	.07	−.15	−.01	.08
Rescue	.05	−.26^*^	−.33^*^	−.28^*^	−.23	−.36^**^	−.12	.12	−.07
SIS Total	.07	.04	.19	.12	.02	.19	.01	−.02	−.02
Planning	.08	.06	.13	.12	.01	.19	.06	−.07	.06
Lethality	.02	.01	.18	.06	.004	.13	−.03	.04	−.09
Clinical impression	.21	.18	.24^*^	.02	.02	.34^**^	−.18	−.17	.13
*Follow-up*									
RRRS Ratio (N=12)	−.07	−.24	.36	−.35	.01	.39	−.33	.04	−.08
Risk	.18	−.36	.28	−.22	.05	.09	−.49	.28	−.34
Rescue	.33	.09	−.19	.43	−.14	−.43	.11	.25	−.18
SIS Total (N=12)	−.31	−.33	−.34	−.22	−.34	.25	.13	.05	.75^**^
Planning	−.29	−.33	−.10	−.19	−.14	.42	.12	.11	.65^*^
Lethality	−.30	−.29	−.52	−.21	−.49	.06	.12	−.01	.76^**^
Clinical impression (N=17)	.08	−.08	.24	.09	−.01	.23	−.02	.12	−.03

Pre-DST, Pre-dexamethasone; C, Cortisol; Post-DST, Post-dexamethasone; RRRS, Risk-Rescue Rating Scale; SIS, Suicidal Intent Scale.

*p < .05; **p < .01.

The adjusted regression analyses confirmed the negative relationship between the rescue sub-score of the RRRS and the pre-DST cortisol value at 9 p.m. (R^2^ =.15, β = −.39, p < .004, CI 95% [− 6.29, − 1.23]), and the post-DST cortisol values at 8a.m. (R^2^ =.17, β = −.32, p < .013, CI 95% [− 6.16, −.76]), and 9 p.m. (R^2^ =.24, β = −.41, p < .001, CI 95% [− 6.89, − 1.76]). They also confirmed the positive relationship between the CGI score and the post-DST cortisol value at 9 p.m. (R^2^ =.19, β =.27, p < .027, CI 95% [.12, 2.04]).

### Sociodemographic and Clinical Characteristics of Individuals Who Attempted Suicide Again During the Follow-Up

During the 2-year follow-up, 26 patients attempted suicide again (n=11 one suicide attempt, and n=15 two or more suicide attempts). Among these 26 patients, only two committed a severe or violent suicide attempt. Moreover, two patients completed suicide during the follow-up, after having attempted suicide at least another time after inclusion. Re-attempters were younger and more often on mood stabilizers than non-re-attempters (n=40) (t63 = 2.53, p < .014; χ2 = 4.10, p < .043, respectively) (**Table 2**). Therefore, logistic regression analyses were adjusted for age and mood stabilizer intake.

### Association Between DST Response and New Suicide Attempt(s) During the Follow-Up

Higher pre-DST cortisol and lower cortisol Δ index values at 8 a.m. predicted higher odds of a new suicide attempt during the 2-year follow-up (β = .76, p < .023, OR [95% CI] = 2.15 [1.11, 4.15], and β = - 2.76, p <.052, OR [95% CI] = .06 [.004, 1.02], respectively)** ^2^ **.

After removing the two suicide completers from the sample, higher pre-DST cortisol (β = .76, p < .026, OR [95% CI] = 2.14 [1.09, 4.18]) and lower cortisol Δ index values at 8 a.m. (β = - 3.00, p < .047, OR [95% CI] = .05 [.003, .96]) remained associated with higher odds of a new suicide attempt.

### DST Response and New Suicide Attempt Features

During the follow-up, the cortisol Δ index at 9 p.m. was positively associated with the total (r = .75, p < .008), planning sub-scale (r = .65, p < .030) and lethality sub-scale scores (r = .76, p < .007) of the SIS. Conversely, the CGI and RRRS scores for the new suicide attempt (or most severe new attempt) during the follow-up were not correlated with the DST response (p > .05) (**Table 4**).

However, the adjusted regression analyses, using the SIS scores as dependent variable and cortisol Δ index values as predictors, retained only the positive relationship between the SIS lethality sub-scale score and Δ index at 9p.m. (R^2^ =.58, β =.86, p < .045, CI 95% [1.12, 73.61]).

## Discussion

This study shows that salivary cortisol is a good predictor of the risk, severity and lethality of suicidal acts at baseline and during the follow-up. Specifically, post-DST cortisol level at 9 p.m. was higher in the violent/serious suicide attempter group than in the non-violent/non-serious attempter group at inclusion. Moreover, post-DST cortisol level at 9 p.m. was related to lower rescue (RRRS) and higher CGI (suicidal severity) scores. We could not test the predictive value of this measure during the follow-up because among the 26 patients who attempted suicide again, only two performed a violent/severe suicidal act. Higher pre-DST cortisol level (significant) and lower Δ index at 8a.m. (trend) predicted higher risk of new suicide attempt in the following 2 years. Finally, the cortisol Δ index value at 9 p.m. was positively correlated with the intent of the most severe suicidal attempt during the follow-up. All these results show the usefulness of salivary cortisol quantification after DST to differentiate among suicide attempt phenotypes and to predict severe/violent suicide attempts.

Although several studies demonstrated that post-DST cortisol level is a good predictor of future suicide completion (12, 13, 15–18), most of the previous works using DST failed to predict suicide attempt at baseline (32, 43, 44) or new attempts (23, 24). Similarly, our results show that post-DST cortisol did not predict future suicide attempts in a sample of patients with history of suicide attempts. However, they show that violent/serious attempters at inclusion had higher post-DST cortisol at 9 p.m. than the other suicide attempters. This is in agreement with previous findings showing non-suppression at the DST in patients with previous serious or violent suicide attempts (23, 30, 31). Regarding the time of day, it is likely that non-violent/non-serious suicide attempters rapidly recovered the normal circadian rhythm after DST, unlike violent/serious attempters. Moreover, patients with lower rescue possibilities (RRRS) and higher suicide attempt severity (CGI score) had higher post-DST cortisol level. A study showed that non-suppressors at the DST are more prone to make a psychologically, not medically serious suicide attempt during the follow-up (31). However, in a 5-year prospective study on 42 patients, Roy (30) did not find any significant difference between patients who attempted suicide with a violent method and with other methods. Thus, it seems that HPA axis dysregulation is related to suicidal characteristics closer to suicide completion. It is now important to determine whether HPA axis dysregulation predicts lifetime riskier attempts or whether it is a short-term consequence after a suicide attempt.

Furthermore, our results show that higher pre-DST cortisol levels at 8a.m. predicted suicide re-attempt. Jokinen et al. (45) found a negative relationship between baseline cortisol (sample obtained in the morning) and suicide attempt during a 20-year follow-up. This difference could be explained by the fact that we included only suicide attempters, and that our follow-up lasted only 2 years. Previous studies reported that higher cortisol awakening response (CAR) is related to higher hopelessness (46) and higher engagement in non-suicidal self-injuries (47), two behaviors that are strongly associated with suicide. Although the pre-DST cortisol level at 8 a.m. is not a measure of CAR, it might partially reflect the morning response. It would be interesting to assess whether CAR is a predictor of past or future suicide attempts.

Finally, when evaluating the changes (Δ index) in post-DST–pre-DST cortisol levels, lower Δ index at 8 a.m. predicted new suicide attempt(s), whereas higher Δ index at 9 p.m. was related to higher suicidal intent. However, after adjusting for confounders only the relationship with the SIS lethality sub-scale score remained significant. This could be explained by the lack of statistical power because of the small number of patients who attempted suicide again during the follow-up. Higher post-DST cortisol level has been related to lower suicide intent at baseline (31), but not during the follow-up. The relationship between cortisol and follow-up SIS score should be interpreted with caution because of the small sample. Yet, these findings show the importance of comparing post-DST and pre-DST levels.

This study has some limitations. First, we did not evaluate current life stressors. Moreover, we used saliva and not serum samples to measure cortisol, although blood sampling is the most common procedure for DST. However, previous studies showed that saliva and blood cortisol measures after DST are highly correlated and can equally predict psychiatric conditions (48, 49). Saliva cortisol testing is less invasive and easier to obtain, giving more opportunities for assessing the DST response in different contexts. Another limitation is the use of two different methods (RIA and ECLIA) to quantify salivary cortisol. Finally, the sample size was small, and the included patients were all recent suicide attempters. This might limit the generalization of our results.

To conclude, we found that DST might be a good test to predict suicide attempts with characteristics of danger for the patient’s life. More research is needed to understand whether higher cortisol levels are the consequence of the suicide attempt severity, or vice versa. Our results also show that post-DST cortisol and pre-DST cortisol levels can predict higher intent and lethality of future suicide attempts, suggesting that cortisol levels could be a predictor of suicide lethality. Finally, higher morning baseline cortisol predicts new suicide attempt(s). Our study adds more evidence to the hypothesis of HPA axis dysregulation in suicide attempters, and highlights the importance of cortisol as a predictive biomarker for suicide.

## Data Availability Statement

All datasets generated for this study are included in the article/**Supplementary Material**.

## Ethics Statement

The studies involving human participants were reviewed and approved by CPP Montpellier Sud-Méditerranée IV, CHU Montpellier. The patients/participants provided their written informed consent to participate in this study.

## Author Contributions

Recruiting participants: AC, CG, EO, PC, SG. Performed the psychiatric interviews: CG, EO, SG, PC. Supervised dexamethasone procedure: AC, CG. Reviewing specific literature: AA-C, CG, IC. Formulating the problem and hypothesis: AA-C, EO, IC, PC, SG. Analyzed data: AA-C. Wrote the paper: AA-C, CG, EO, SG, PC. Approved the final version of paper: AA-C, AC, CG, EO, IC, PC, SG.

## Funding

This study was financially supported by Programme Hospitalier de Recherche Clinique (CHU of Montpellier- PHRC UF 7653).

## Conflict of Interest

The authors declare that the research was conducted in the absence of any commercial or financial relationships that could be construed as a potential conflict of interest.

The handling editor declared a past co-authorship with several of the authors CG, SG, PC.

## Footnotes

1 See Figure 2 from Kaseva et al. (10) for a summary of the salivary cortisol response in healthy people the day before and after the DST.

2 57 of the 70 patients completed the childhood trauma questionnaire [CTQ: Bernstein et al., 42]. This questionnaire was not included in the main analysis due to the reduction of statistical power. The distribution of childhood trauma or neglect was not significantly different (all p > .05) between violent/serious *vs* non-violent/non-serious suicide attempters and also between re-attempters *vs* non- re-attempters during the follow-up. Conversely, pre-DST cortisol at 8 a.m. was positively correlated with physical neglect (r =.22, p < .05); no other significant correlation –was identified (all p > .05). A sensitivity analysis was performed to investigate the relationship between pre-DST cortisol at 8 a.m. and reattempt during the follow-up after adjusting for physical neglect. This relationship remained significant (β = 1.14, p < .006, OR [95% CI] = 3.12 [1.39, 6.99]).
